# Clonogenic assays improve determination of variant allele frequency of driver mutations in myeloproliferative neoplasms

**DOI:** 10.1007/s00277-022-05000-9

**Published:** 2022-10-21

**Authors:** Milena Kalmer, Kristina Pannen, Rebecca Lemanzyk, Chiara Wirths, Julian Baumeister, Angela Maurer, Kim Kricheldorf, Joelle Schifflers, Deniz Gezer, Susanne Isfort, Tim H. Brümmendorf, Steffen Koschmieder, Nicolas Chatain

**Affiliations:** 1 Department of Hematology, Oncology, Hemostaseology, and Stem Cell Transplantation, Faculty of Medicine, RWTH Aachen University, Pauwelsstraße 30, 52074 Aachen, Germany; 2 Center for Integrated Oncology, Aachen Bonn Cologne Düsseldorf (CIO ABCD), Aachen, Germany

**Keywords:** Triple negative MPN, Clonogenic assay, Next-generation sequencing (NGS), Driver mutations, Variant allele frequency (VAF), Lactate dehydrogenase (LDH), JAK2V617F, CALR, MPL

## Abstract

Molecular diagnostics moves more into focus as technology advances. In patients with myeloproliferative neoplasms (MPN), identification and monitoring of the driver mutations have become an integral part of diagnosis and monitoring of the disease. In some patients, none of the known driver mutations (*JAK2*V617F, *CALR*, *MPL*) is found, and they are termed “triple negative” (TN). Also, whole-blood variant allele frequency (VAF) of driver mutations may not adequately reflect the VAF in the stem cells driving the disease. We reasoned that colony forming unit (CFU) assay–derived clonogenic cells may be better suited than next-generation sequencing (NGS) of whole blood to detect driver mutations in TN patients and to provide a VAF of disease-driving cells. We have included 59 patients carrying the most common driver mutations in the establishment or our model. Interestingly, cloning efficiency correlated with whole blood VAF (*p* = 0.0048), suggesting that the number of disease-driving cells correlated with VAF. Furthermore, the clonogenic VAF correlated significantly with the NGS VAF (*p* < 0.0001). This correlation was lost in patients with an NGS VAF <15%. Further analysis showed that in patients with a VAF <15% by NGS, clonogenic VAF was higher than NGS VAF (*p* = 0.003), suggesting an enrichment of low numbers of disease-driving cells in CFU assays. However, our approach did not enhance the identification of driver mutations in 5 TN patients. A significant correlation of lactate dehydrogenase (LDH) serum levels with both CFU- and NGS-derived VAF was found. Our results demonstrate that enrichment for clonogenic cells can improve the detection of MPN driver mutations in patients with low VAF and that LDH levels correlate with VAF.

**Supplementary Information:**

The online version contains supplementary material available at 10.1007/s00277-022-05000-9.

## Introduction

Myeloproliferative neoplasms (MPN) comprise a group of chronic hematological malignancies [1]. The majority of patients with Philadelphia chromosome-negative MPN carry one of the common driver mutations in the genes of *JAK2* (*JAK2*V617F), calreticulin (*CALR*del52, *CALR*ins5), or the thrombopoietin receptor (*MPL*W515L/K) [2–4]. These driver mutations can be found in 85–90% of MPN patients, while 10–15% of patients are termed “triple negative” (TN), due to the lack of the most abundant mutations found in these three genes [5]. Current personalized medicine relies on the identification of driver mutations in every patient, as progression of the disease may differ and therapy may need to be adjusted depending on the mutation load [6].

The introduction of next-generation sequencing (NGS) and the analysis of the patients' genetic landscape in routine diagnostics have improved the understanding of the impact of genetic alterations on prognosis. Previously, different methods of analysis were applied with very different sensitivities. In the past, Sanger sequencing was primarily used, but the technology is limited by a relatively low detection threshold of 5–20% mutant allele burden or variant allele frequency (VAF) [7, 8]. Considerably higher sensitivity was described for allele-specific PCR [7, 8] or qPCR [9] — down to 0.01% VAF—, but these methods can only detect a single mutation, as specific primers are necessary. In comparison, NGS is a robust method able to simultaneously detect a large number of mutations [10] down to a VAF of 2% or lower [11]. Nevertheless, a fraction of patients remains TN.

Therefore, we investigated whether identification of driver mutations can be improved using colony forming unit (CFU) assays. In these assays, peripheral blood mononuclear cells (PBMC) are seeded in semi-solid medium, each grown colony representing one single cell that has the potential to proliferate. MPNs are clonal diseases, and we hypothesized that specifically mutated cells show a growth advantage and that the CFU assay enriches mutated disease–driving progenitors. By genotyping individual colonies, we aimed to find mutated alleles that could not be detected by analyzing non-enriched PBMCs.

## Materials and methods

PB samples from MPN patients were collected at the Department of Hematology, Oncology, Hemostaseology and Stem Cell Transplantation at RWTH Aachen University Medical Center after patients’ written informed consent, as approved by the local ethics committee (EK 127/12). PBMCs from MPN patients were isolated via density-gradient centrifugation (Ficoll; GE Healthcare, USA, or Pancoll; Pan Biotech, Germany). Cells were seeded at a density of 1 × 10^6^ cells/ml methylcellulose (StemCell Technologies, Canada) for polycythemia vera (PV) and essential thrombocythemia (ET) patients, while 2.5-5 × 10^5^ cells/ml were applied for myelofibrosis (MF) samples due to the higher expected cloning efficiency of MF progenitor cells. Methylcellulose (StemCell Technologies, Canada) was supplemented with 20 ml IMDM (Gibco, USA) and 50 ng/ml hSCF, 10 ng/ml hIL-3, 10 ng/ml hGM-CSF, and 14 ng/ml hEPO (all Immunotools, Germany). After 10-14 days, distinct colonies were counted and the percentage of colony forming cells (CFC) or cloning efficiency was calculated by dividing the number of grown colonies by the number of cells that was seeded. 25-30 colonies each were individually picked and lysed in RLT buffer (Qiagen, Germany) followed by the isolation of the genomic DNA using the PCR and DNA Cleanup Kit (New England Biolabs, USA). The DNA of the individual colonies was then screened for mutations by allele-specific PCR. The type of colony analyzed depended on the disease, PV-derived colonies presented in the majority as CFU-E or BFU-E, while in PMF patients, white colonies prevailed. In ET patients, both types of colonies grew; therefore, both were picked.

For each colony, an allele-specific PCR for *JAK2*V617F or *JAK*2WT was performed. In addition, a *CALR* PCR, which comprises the common deletions in exon 9 (e.g., del52), was performed, and the difference in size of the resulting DNA fragments between mutant and WT *CALR* was analyzed. For the analysis of *CALR*ins5 and *CALR*WT, an allele-specific PCR was applied. For the analysis of *MPL*W515K and W515L, two individual allele specific PCRs were established based on the study of Takei and colleagues [12], using primary patient DNA that had already been analyzed using NGS. The protocol was then applied to single colonies. A list of all primers used in this study is provided in Supplemental table S1. PCR protocols are listed in Supplemental table S2. PCR products were analyzed by agarose gel electrophoresis. Exemplary agarose gels of all performed PCRs are shown in Supplemental Fig. S1.

Following genotyping, the clonogenic VAF was calculated based on the number of mutated alleles found in the colonies, with a WT colony counting as two WT alleles, heterozygous colonies counting as one WT and one mutated allele, and homozygous colonies counting as two mutated alleles.

Routinely, the VAF of all patients was assessed using cells from whole blood after lysis of erythrocytes via NGS as described previously [13] (see list of analyzed genes in the Supplementary Material). Leukocytes, platelets, and hemoglobin (Hb) levels were measured using a Sysmex XS-800i from EDTA blood samples. Serum LDH was determined photometrically as part of the clinical routine. For the generation of all figures and for statistics, GraphPad Prism 9 was used, and Microsoft Excel was used for all calculations.

## Results

### Cloning efficiency is phenotype- and VAF-dependent

**Fig. 1:**
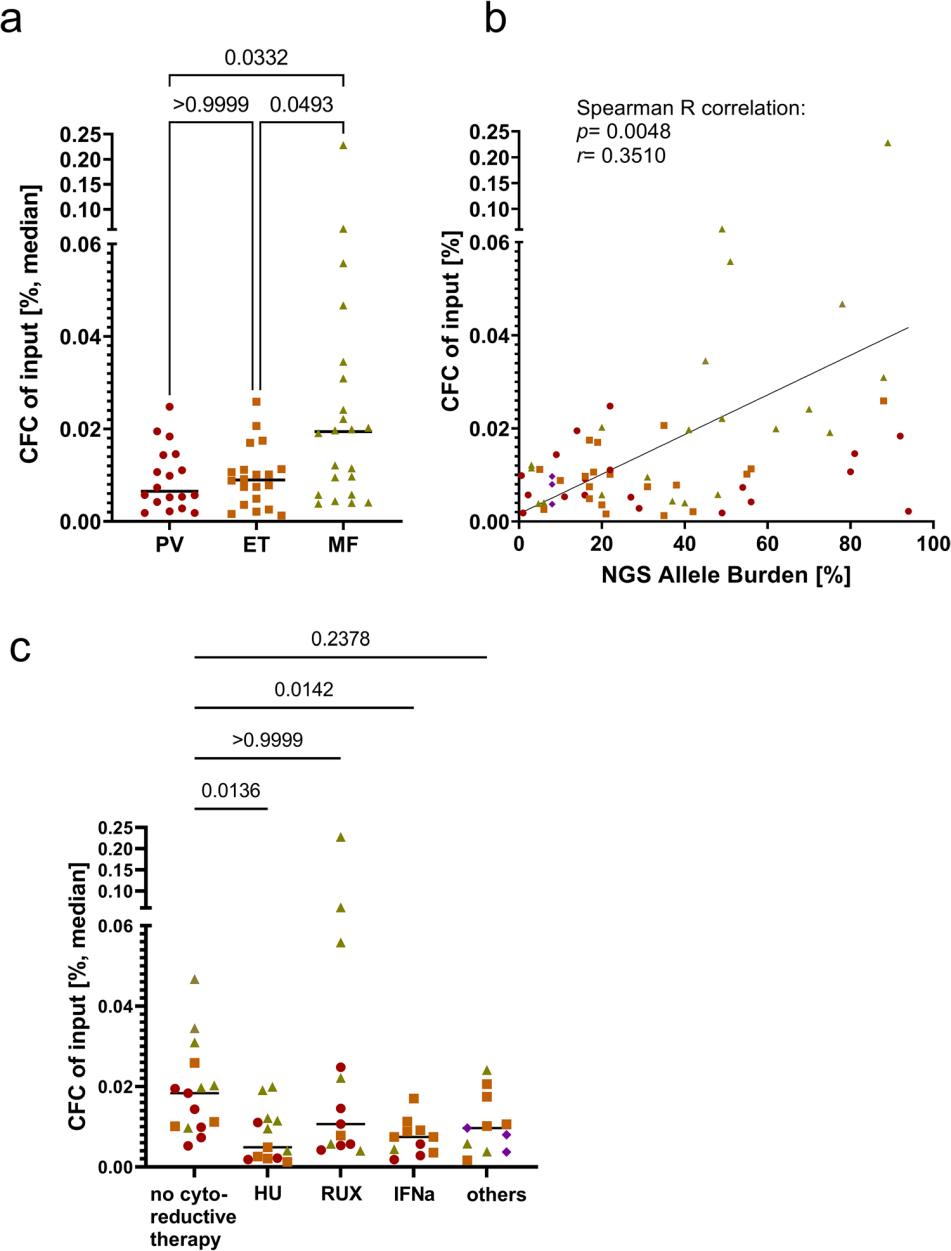
Analysis of cloning efficiency and VAF correlation. **a** PBMCs of MPN patients were seeded in CFU assays, counted after 10–14 days, and the percentage of CFC of input (cloning efficiency) was calculated. All CFUs were grouped by disease subtype. Statistical analysis was done using the Kruskal-Wallis test with Dunn’s multiple comparison. **b** Percentage CFC was correlated with VAF (%) determined via NGS. Correlation was analyzed using the Spearman *R* test. **c** Patients were grouped based on their in vivo treatment. No cytoreductive therapy — no treatment, phlebotomies, and/or ASS; HU, hydroxyurea; RUX, ruxolitinib; IFNa (pegylated), interferon alpha; others — e.g., Imetelstat, anagrelide, or combination treatment. Statistical analysis was done using the Kruskal-Wallis test with Dunn’s multiple comparison. red dots — PV; orange squares — ET; green triangles — MF, violet hashes — MPN-unclassifiable

For the establishment of the method, PBMCs were seeded, and the number of grown colonies was determined 10–14 days after seeding. To analyze whether the cloning efficiency differed between the patients, the percentage of CFC per seeded cells was determined. Here, PV and ET patients presented with a similar percentage of CFC, while this number was elevated in MF patients, as shown in Fig. 1a. A summary of all patients’ NGS VAF and CFC is provided in Supplemental table S3. Next, we analyzed whether the percentage of CFC was correlated with the VAF. The VAF used for this analysis was determined using NGS. Indeed, a significant correlation of VAF and the percentage of CFC was observed (Fig. 1b; *p* = 0.0048 and *r* = 0.3510). We analyzed whether we could find a correlation between CFC and additional mutations, but no such relation was found. Moreover, we analyzed whether treatment of the patients has an influence on the CFC. A significant decrease of CFC was found in hydroxyurea (HU) and interferon alpha (IFNa) treated patients as shown in Fig. 1c, while no significant difference in cloning efficiency was seen when patients were treated with ruxolitinib (RUX). We sought to investigate whether there was a significant correlation of cloning efficiency with the percentage of blasts in the PB. However, 80% of the analyzed patients did not have any blasts at the time of sampling, therefore, there was no correlation between the two parameters (data not shown).

Next, we analyzed the comparability of cloning efficiency of cryo-conserved vs. freshly isolated PBMCs. We cultured fresh vs. previously frozen PBMCs of 5 patients from the same sampling date and compared the resulting CFC. CFC was slightly but not significantly decreased in the frozen samples as shown in Supplemental Fig S2. We therefore concluded that the cloning efficiency of the cells before and after cryo-conservation is comparable to slightly decreased.

### Clonogenic enrichment of immature cells in PB improves the sensitivity of driver mutation detection at low mutational VAF

To analyze how efficient low frequencies of mutated alleles can be detected using genotyping of CFU assays, 50 patients were analyzed, of which 15 had a VAF of 15% or lower according to NGS. Of all patients, 36 were positive for *JAK2*V617F, 6 carried a *CALR*del52 mutation, 5 carried a *CALR*ins5 mutation, and 3 carried *MPL*W515K/L mutations. An overview of all patients analyzed can be found in Supplemental table S3.

**Fig. 2:**
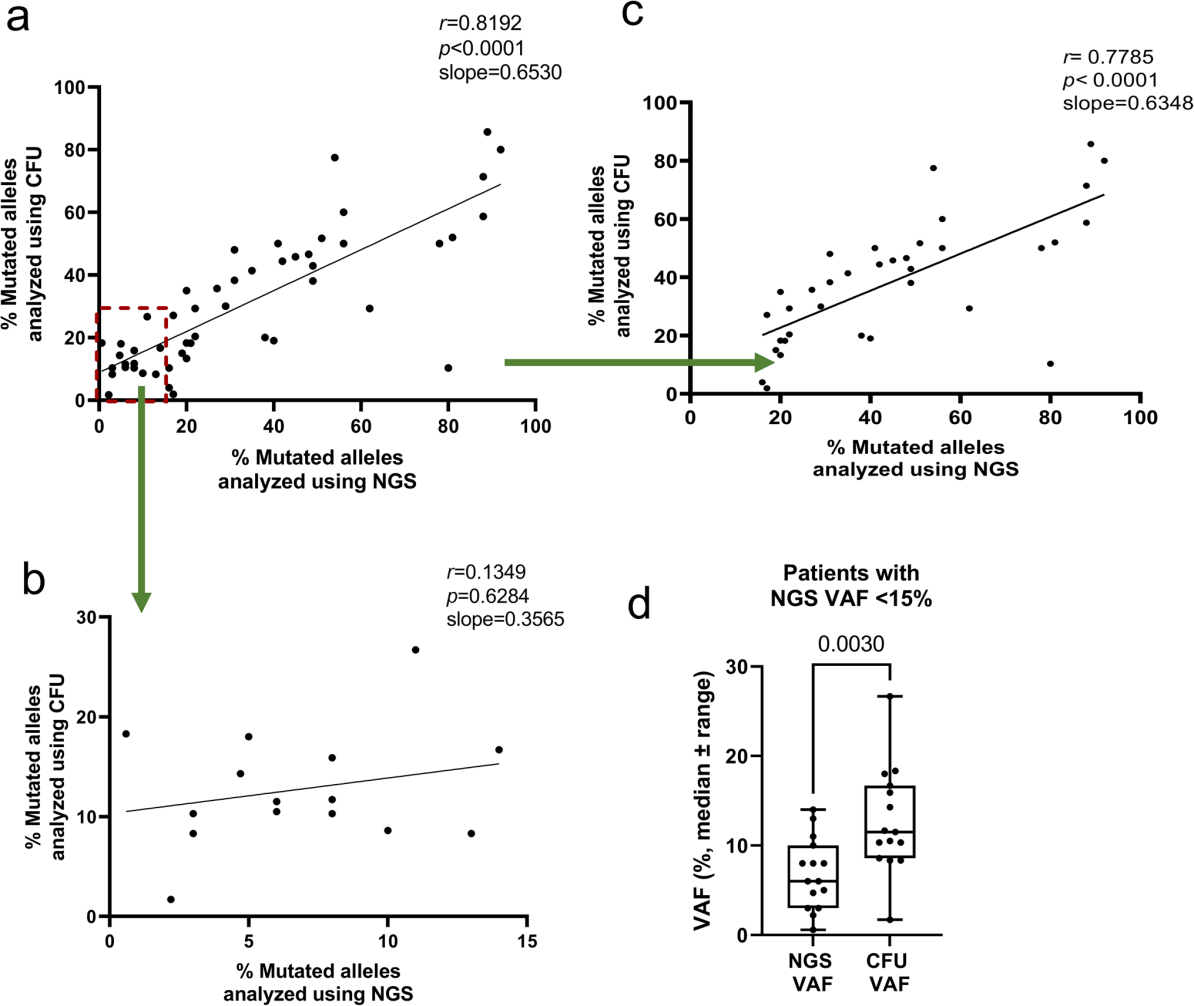
Clonogenic and NGS VAF correlate significantly. Whole blood after lysis of erythrocytes from Ph-MPN patients was analyzed for the presence of driver mutations using NGS. PBMCs of the same patients were isolated and seeded in CFU assays. The clonogenic VAF was determined using genotyping of 30 colonies per patient. **a** All analyzed samples are shown (*n* = 50). Red square marks patients used for analysis of low frequented mutations, shown in **b**. **b** Analysis of patients with a VAF ≤ 15% according to NGS (*n* = 15). **c** Analysis of patients with a VAF ≥ 15% according to NGS (*n* = 35). All correlations were analyzed using the Spearman *R* test. **d** Analysis of different VAFs in patients with NGS VAF < 15%. Statistical analysis was done using the Mann-Whitney test

DNA of individual colonies was isolated, and the corresponding PCR was performed for the mutation found by NGS. More than 70% of picked colonies and isolated DNA successfully yielded a clear band in gel electrophoresis after PCR (Supplemental table S4). Importantly, the clinically reported mutation was confirmed in the colonies of all patients. The calculated clonogenic VAF was lower than the one calculated using NGS of whole blood, but both VAFs showed a highly significant correlation (Fig. 2a; *p* < 0.0001).

Aiming to find low-frequency mutations, we analyzed patients with an NGS-derived VAF of ≤ 15% in more detail (Fig. 2b). Interestingly, there was no significant correlation anymore between the clonogenic and NGS-derived VAFs ≤ 15% (Fig. 2b). This was due to a higher clonogenic VAF in these patients (Fig. 2d), indicating an enrichment of disease-driving cells. Quantification of this effect confirmed that the clonogenic VAF was significantly higher than NGS VAF (*p* = 0.003) in patients with an NGS VAF below 15%, as shown in Fig. 2d. Conversely, in patients with an NGS-derived VAF >15%, we were able to find a significant correlation between the clonogenic and the NGS VAF (Fig. 2c), but, here, the clonogenic VAF was generally lower than the NGS VAF, suggesting that higher VAFs in the PB were driven more by mature cells than by the disease-driving immature cells.

To investigate whether genotyping of samples from the same patient yielded the same VAF, one MPN-U patient was analyzed three times, once using fresh PBMCs, once frozen PBMCs, and once fresh PBMCs from a later time point. The two clonogenic VAF from the same time point (NGS VAF 8%) were calculated with 10.3% and 11.7%, while the later time point yielded 15.9% (Supplemental table S3, patient #9). Additionally, one PMF patient (Supplemental Table S3, patient #4, NGS VAF 3%) was analyzed using fresh and frozen cells from the same timepoint and clonogenic VAF yielded numbers of 8.3 and 10.3%, respectively. This indicates that the method is quite reliable and that frozen cells can be used for the generation of reproducible data.

Additionally, we evaluated whether we could see differences in VAF when comparing different treatments of the patients. No differences were observed due to treatment for the NGS VAF in the different groups, as shown in Supplemental Fig S3a. For the clonogenic VAF, we observed a trend towards a decreased VAF in the treated groups except for the RUX-treated group in comparison to the untreated patients (Supplemental Fig S3b).

### Clonogenic growth does not enhance low copy detection in triple-negative MPN patient material

We hypothesized that we may be able to achieve an enrichment of mutated cells and thereby increase the sensitivity of detecting common driver mutations of low frequency in TN patients. Therefore, PBMCs from 5 TN patients were analyzed by CFU assay. For each colony, five PCRs were performed to detect *JAK2*V617F, deletions in *CALR*, *CALR*ins5, *MPL*W515K, or *MPL*W515L. None of these mutations were detected in any of the colonies, which support the NGS results and the classification of the patients as TN (Table 1).

**Table 1:** Triple-negative (TN) patients and their mutational analysis over time

Patient	Diagnosis	Material	Date of sample	NGS results concerning driver mutation	Additional mutations (NGS)	Qualitative PCR from PBMCs	Clonogenic VAF
TN1	ET	PB	2019	Triple negative	-	2014 triple negative	Triple negative
TN2	PV	PB	2020	Triple negative	-	-	Triple negative
TN3	Post-ET-MF	PB	2017	Triple negative	-	2015 triple negative	Triple negative
		PB	2018	Triple negative	-		
		PB	2019	Triple negative	-		
		PB	2019	Triple negative	-		
		PB	2020	Triple negative	-		
TN4	MPN/MDS Overlap	PB	2017	Triple negative	40% ASXL1 P1377fs, 44% SETBP1 G870S, 31% SRSF2 R94dup	-	Triple negative (2017)
		PB	2020	14% *JAK2* V617F	30% ASXL1 P1377fs, 15% IDH2 R140Q, 8,5% NRAS G13C, 14% NRAS G13D, 28% SETBP1 G870S, 35% SRSF2 R94dup		
TN5	ET	PB	2017	Triple negative	3.8% DNMT3A S714C	-	Triple negative
		PB	2019	Triple negative	6.75% DNMT3A S714C		

One of the analyzed MPN patients had been grouped as being TN in 2017 via NGS (Table 1; patient TN4); the CFU assay was performed in 2017 and confirmed the absence of driver mutations. Additionally, we retrospectively analyzed the DNA used for NGS with an allele specific PCR and were not able to detect the *JAK2*V617F mutation in this sample (data not shown). This patient was reanalyzed via NGS in 2020, and a *JAK2*V617F VAF of 14% was determined. Thus, the clone carrying the mutation may have already been present in 2017 but had not expanded to a detectable level. In this case, using CFU, we were not able to observe an enhancement of the detection limit. Alternatively, the patient may have acquired the *JAK2*V617F mutation after 2017.

One *CALR*del52-mutated patient with a VAF of 16% was included into this study (Supplemental Table S3; patient #15). Initially, this patient was categorized as being TN in 2014 using standardized PCRs. In 2017, a *CALR*del52 mutation was detected via NGS. Comparable to patient TN4, it is possible that the mutated clone developed in the meantime. PBMCs of patient #15, isolated in 2017, were also used in the CFU assay and the *CALR* mutation was successfully detected.

### Clonogenic and NGS VAF correlate with LDH levels

**Fig. 3:**
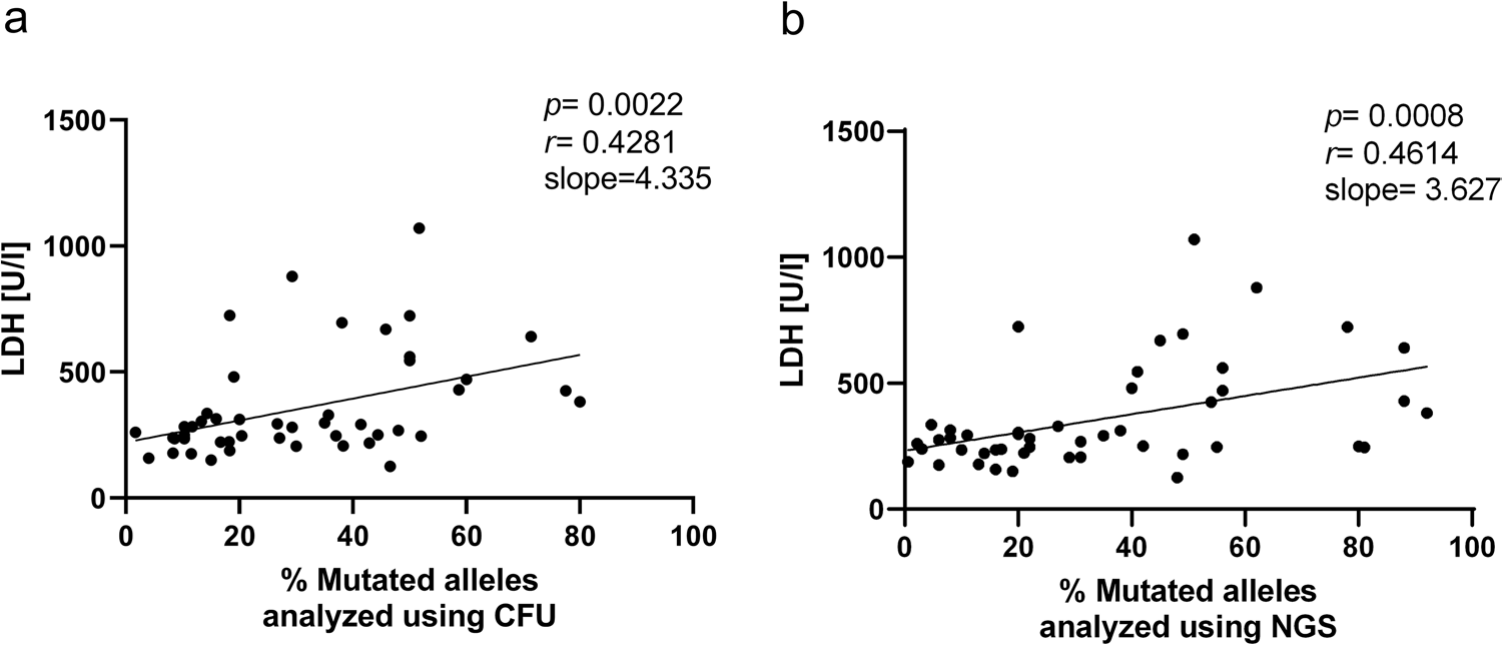
LDH levels correlated significantly with mutant VAF. **a** Correlation of LDH levels with clonal VAF in all patients. **b** Correlation of LDH levels with NGS VAF in all patients. All correlations were analyzed using the Spearman *R* test

Next, we correlated markers that are used for monitoring disease progression with the clonogenic and the NGS-derived VAF to further validate our data. LDH is a maker for cell turnover, and elevated levels were already described to be associated with adverse survival [14]. LDH levels correlated with both the clonogenic (Fig. 3a) and the NGS VAF (Fig. 3b). Comparison with the clonogenic VAF was highly significant (*p* = 0.0022, *r* = 0.4281) with a slope of 4.335. In comparison, LDH significantly correlated with NGS VAF (*p =* 0.0008, *r* = 0.4614) with a slope of 3.627. Both methods therefore yield very similar results confirming the validity of both VAF determinations. However, no correlation was found between VAF and platelets, hemoglobin, or leukocytes (Supplemental Fig. S4a-f).

## Discussion

Identification of driver mutations and development of mutant VAF increasingly moves into the focus of MPN diagnostics due to the specific tailoring of treatment for each patient. Therefore, the aim of this study was to investigate whether the sensitivity of driver mutation detection can be increased using CFU assays of PBMCs compared to NGS analysis of whole blood. While for the analysis of whole blood, growth rates of specific clones cannot be dissected, individual clones can be analyzed in single colonies.

First, we observed that CFC numbers in MF patients were significantly increased compared to PV and ET patients. This result indicates a higher colony forming potential for circulating cells from MF patients. In addition, through progressing fibrosis and enhanced extramedullary hematopoiesis, more progenitor-like cells are mobilized from the BM to the peripheral blood or spleen. In line with our data, Andréasson and colleagues described increased levels of CD34+ cells in PB from myelofibrosis patients in comparison to PV and ET [15]. Additionally, a significant correlation of the VAF and the CFC was observed. This indicates that mutated cells have acquired a higher growth potential than WT cells and that due to their increased fraction in patients with high VAF, a higher cloning efficiency was observed. Furthermore, a decrease in CFC was observed in patients treated with HU and IFNa, but not in patients treated with RUX, potentially indicating suppression of progenitor cells by HU and IFNa but not consistently by RUX.

We clearly show that genotyping of single colonies recapitulated the detection of mutations that had previously been found via NGS of whole blood, even in patients with very low VAF. The calculated VAF using the CFU, although slightly lower, correlated with the VAF determined via NGS. The lower clonogenic VAF is not entirely surprising, as two different methods of analyses will never give the exact same value. Furthermore, the cytokines in the methylcellulose may promote growth of WT cells, while the mutated ones are not dependent but also not as susceptible explaining lower VAF calculated using the CFU assay. Lastly, for NGS analysis, whole blood was used, while in the CFU assay, each colony resembles a single clone, going back to a primitive cell that still contains some stemness. As the granulocytic compartment can be involved in the MPN-phenotype and mutated cells have increased growth rates, NGS results could give a distorted picture of the VAF as the diseased cells are enriched here, which has been described by Van Egeren et al. [16]. This can be circumvented by analyzing the clonogenic VAF as the size of the colonies does not influence the calculation of the VAF, therefore reflecting the stem cell compartment more adequately.

However, when analyzing the patients with a VAF <15% in more detail, we observed that the correlation is lost and the clonogenic VAF was significantly higher than the NGS VAF. This indicates that we achieve an enrichment of low-frequency mutations in colony assays. It is possible that in these patients, the ratio of MPN driver cells was not high enough to lead to changes in the microenvironment, which promote a high proliferation of MPN cells. Recent studies have investigated the time between the initial occurrence of driver mutations in MPN and the initiation of the disease [16, 17]. They found that mutated cells can be traced back to early childhood or even in utero and that the mean duration until initiation of overt clinical disease was around 30 years. This shows that from the event of the mutation until the detectable expansion of MPN cells, additional events are necessary, which we observed only in patients with a VAF above 15% (Supplementary Table S3).

A potential pitfall of our method is the analysis of the limited number of colonies per patient, 30 in our assay, which means that each allele corresponds to 1.67% of the calculated VAF (2 alleles per colony, 30 colonies per patient, 60 alleles in total, which are used for the calculation). Therefore, each allele influenced the calculated VAF strongly, and the calculation may not be sensitive enough when only a low number of mutated alleles are present.

As it has recently been reported that mutated clones can exist decades before the disease becomes clinically apparent [16, 17], we sought to determine whether it is possible to detect driver mutations in TN patients by enriching for MPN driver cells in a CFU assay. In the five TN patients included in our study, no mutations were found by genotyping of colonies. This might be due to the true absence of the most common driver mutations we screened for or due to the low number of mutated cells in these patients. In the case of the latter, genotyping of more colonies might be an opportunity to be able to recover mutated clones, even if the number is very small. In comparison to NGS analysis, CFU assays did not increase the sensitivity of mutated allele detection.

Apart from monitoring the VAF, blood parameters such as complete blood counts and serum LDH are routinely measured in MPN patients. In our study, we have evaluated four of these parameters (leukocytes, Hb, platelets, and serum LDH) and investigated whether they correlate with the VAF. No such correlations were found for platelets, Hb, or leukocytes, which was not surprising as these patients are already being treated, and while a hematologic response is often achieved, a molecular response is rare or depending on the treatment [18, 19]. Additionally, LDH levels were determined as they can be used as an indicator of cell turnover and have been reported to be elevated in PV, ET, and MF patients [14, 20, 21]. Here, a correlation between the LDH levels and the VAF was found. However, there was no difference between the methods used for the determination of the VAF.

The enrichment of the clonogenic VAF in patients with a low NGS VAF raises the question of whether determination of the clonogenic VAF is a feasible diagnostic tool. We propose that it might be a helpful additional tool in certain cases, as it may provide additional information of the amount of mutated cells in the early stem and progenitor cell compartment, especially at the onset of the disease when the VAF is still low. However, the procedure is limited by its costs and labor-intensive workflow and may thus not be suitable for routine diagnostics.

In conclusion, we have demonstrated a strong correlation of CFU- and NGS-derived VAF for MPN driver mutations in the PB in patients with VAF >15%. However, CFU-derived VAF detection was superior to NGS-derived VAF in patients with VAF ≤15%, suggesting enrichment for MPN-driving cells by clonogenic analysis.

## Supplementary Information

Below is the link to the electronic supplementary material.

Supplementary file1 (DOCX 731 KB)

## Abbreviations

CFC: colony forming cells
CFU: colony forming unit
ET: essential thrombocythemia
Hb: hemoglobin
HU: Hydroxyurea
IFNa: interferon alpha
LDH: lactate dehydrogenase
MF: myelofibrosis
MPN: myeloproliferative neoplasms
NGS: next-generation sequencing
PBMC: peripheral blood mononuclear cells
PV: polycythemia vera
RUX: Ruxolitinib
TN: triple negative
VAF: variant allele frequency
WT: wildtype
