# Supplementary material for: Clonogenic assays improve determination of variant allele frequency of driver mutations in myeloproliferative neoplasms
Source: Ann Hematol. 2022 Oct 21;101(12):2655–63. doi: 10.1007/s00277-022-05000-9 (PMC9646600; doi:10.1007/s00277-022-05000-9)
Supplement: Supplementary file 1 — Supplementary file1 (DOCX 731 KB) [file 277_2022_5000_MOESM1_ESM.docx]

# Supplemental material

# Annals of Hematology

# Clonogenic assays improve determination of variant allele frequency of driver mutations in myeloproliferative neoplasms

Milena Kalmer^1,2^, Kristina Pannen^1,2^, Rebecca Lemanzyk^1,2^, Chiara Wirths^1,2^, Julian Baumeister^1,2^, Angela Maurer^1,2^, Kim Kricheldorf^1,2^ , Joelle Schifflers^1,2^, Deniz Gezer^1,2^, Susanne Isfort^1,2^, Tim H. Brümmendorf ^1,2^, Steffen Koschmieder^1,2,3^, Nicolas Chatain^1,2,3,4^

^1^Department of Hematology, Oncology, Hemostaseology, and Stem Cell Transplantation, Faculty of Medicine, RWTH Aachen University, Pauwelsstraße 30, 52074 Aachen, Germany

^2^Center for Integrated Oncology Aachen Bonn Cologne Düsseldorf (CIO ABCD), Aachen, Germany

^3^contributed equally

^4^corresponding author

Corresponding Author:

Dr. rer. nat. Nicolas Chatain, Department of Hematology, Oncology, Hemostaseology, and Stem Cell Transplantation, Faculty of Medicine, RWTH Aachen University, Pauwelsstr. 30, D-52074 Aachen, Germany, Phone: +49-241-8037798; E-mail: nchatain@ukaachen.de

**Supplemental Table S1 Primer sequences**

| **Name** | **Sequence** |
| --- | --- |
| JAK2 forward | TCC TCA GAA CGT TGA TGG CAG |
| JAK2 reverse | ATT GCT TTC CTT TTT CAC AAG AT |
| JAK2 mut | GTT TTA CTT ACT CTC GTC TCC ACA AAA |
| JAK2 WT | GCA TTT GGT TTT AAA TTA TGG AGT ATA TG |
| CALR forward | ACA ACT TCC TCA TCA CCA ACG |
| CALR reverse | GGC CTC AGT CCA GCC CTG |
| CALR ins5 forward | TAA CTG CAG TGT CAG CGG TG |
| CALR WT reverse | TGT CCT CAT CAT CCT CCT TG |
| CALR ins5 reverse | TGT CCT CAT CAT CCT CCG AC |
| MPL forward | TGA CCG CTC TGC ATC TAG TGC |
| MPL reverse | GGT CAC AGA GCG AAC CAA GA |
| MPL W515L forward | GGC CTG CTG CTG CTG AAG TT |
| MPL W515K reverse | ACC TGT AGT GTG CAG GAA ACT TCT T |

**Supplemental Table S2 Details of different PCRs**

| **JAK2 WT** |  |
| --- | --- |
| REDTaq^®^ DNA-Polymerase |  |
| dNTP-Mix | 150 µM |
| Primer JAK2 reverse | 100 nM |
| Primer JAK2 WT | 50 nM |
| Annealing Temperature | 55 °C |

| **JAK2 Mut** |  |
| --- | --- |
| REDTaq^®^ DNA-Polymerase |  |
| dNTP-Mix | 150 µM |
| Primer JAK2 forward | 100 nM |
| Primer JAK2 mut | 50 nM |
| Annealing Temperature | 55 °C |

| **CALR** |  |
| --- | --- |
| DreamTaq Green PCR Master Mix | |
| Primer CALR forward | 40 nM |
| Primer CALR reverse | 40 nM |
| Annealing Temperature | 64.5 °C |

| **CALR ins5** |  |
| --- | --- |
| DreamTaq Green PCR Master Mix | |
| Primer CALR ins5 forward | 10 nM |
| Primer CALR ins5 reverse | 10 nM |
| Annealing Temperature | 60 °C |

| **CALR WT** |  |
| --- | --- |
| DreamTaq Green PCR Master Mix | |
| Primer CALR ins5 forward | 10 nM |
| Primer CALR WT reverse | 10 nM |
| Annealing Temperature | 60 °C |

| **MPLW515L** |  |
| --- | --- |
| DreamTaq Green PCR Master Mix | |
| MPL reverse | 100 nM |
| MPL W515L forward | 8 µM |
| Annealing Temperature | 59 °C |

| **MPLW515K** |  |
| --- | --- |
| DreamTaq Green PCR Master Mix | |
| MPL forward | 100 nM |
| MPL W515K reverse | 240 nM |
| Annealing Temperature | 59 °C |

**Supplemental experimental procedures**

**List of genes analyzed in NGS**

Either 31 (ABL1, ASXL1, BARD1, CALR, CBL, CHEK2, CSF3R, DNMT3A, ETNK1, ETV6, EZH2, IDH1, IDH2, JAK2, KIT, KRAS, MPL, NFE2, NRAS, PDGFRA, PTPN11, RUNX1, SETBP1, SF3A1, SF3B1, SH2B3 (LNK), SRSF2, TCF12, TET2, TP53, U2AF1)(Kirschner et al., 2018) or 32 genes (ABL1, ASXL1, BRAF, BTK, CALR, CBL, CSF3R, CXCR4, DNMT3A, ETNK1, EZH2, FLT3, IDH1, IDH2, JAK2, KIT, KRAS, MPL, MYD88, NFE2, NPM1, NRAS, PTPN11, RUNX1, SETBP1, SF3B1, SH2B3, STAT5B, TET2, TP53, U2AF1, WT1) were analyzed in each sample.

**Supplemental Table S3**

| **Patient #** | **Diagnosis** | **NGS VAF [%]** | **Driver mutation** | **% CFC** | **Treatment** | **Additional mutations** | **% of genotyped colonies** | **Clonogenic VAF [%]** | **Platelets [/nl]** | **Hemoglobin [g/dl]** | **Leukocytes [/nl]** | **LDH [U/l]** |
| --- | --- | --- | --- | --- | --- | --- | --- | --- | --- | --- | --- | --- |
| 1 | PV | 0.59 | *JAK2*V617F | 0.00987 | no cytoreductive therapy | - | 100.00 | 18.33 | 228 | 17.3 | 5.9 | 188 |
| 2 | PV | 1.00 | *JAK2V617F* | 0.00183 | IFNa | - | n.a. | n.a. | n.a. | n.a. | n.a. | n.a. |
| 3 | PV | 2.20 | *JAK2*V617F | 0.0057 | RUX | - | 100.00 | 1.70 | 252 | 8.8 | 6.2 | 260 |
| 4 | PMF | 3.00 | *JAK2*V617F | 0.0115 | HU | - | 100.00 | 8.33 | 856 | 15.3 | 16.7 | 239 |
|  | PMF | 3.00 | *JAK2*V617F | 0.01213 | HU | - | 96.67 | 10.34 | 856 | 15.3 | 16.7 | 239 |
| 5 | PMF | 4.70 | *JAK2*V617F | 0.003838 | others | - | 70.00 | 14.29 | 352 | 6.0 | 14.3 | 336 |
| 6 | ET | 5.00 | *JAK2*V617F | 0.011184 | no cytoreductive therapy | - | 100.00 | 18.00 | n.a. | n.a. | n.a. | n.a. |
| 7 | ET | 6.00 | *JAK2*V617F | 0.002583 | HU | - | 92.00 | 10.50 | 405 | 13.4 | 7.8 | 276 |
| 8 | PMF | 6.00 | *JAK2*V617F | 0.004061 | HU | - | 52.00 | 11.50 | 343 | 13.9 | 5.5 | 176 |
| 9 | MPNu | 8.00 | *JAK2*V617F | 0.003739 | others | - | 100.00 | 10.34 | 933 | 14.9 | 7.6 | 283 |
|  | MPNu | 8.00 | *JAK2*V617F | 0.008 | others | - | 88.00 | 11.67 | 933 | 14.9 | 7.6 | 283 |
|  | MPNu | 8.00 | *JAK2*V617F | 0.0097 | others | - | 96.67 | 15.90 | 616 | 14.2 | 6.7 | 314 |
| 10 | PV | 9.00 | *JAK2V617F* | 0.01437 | no cytoreductive therapy | - | n.a. | n.a. | n.a. | n.a. | n.a. | n.a. |
| 11 | ET | 10.00 | *CALR*del52 | 0.008824 | IFNa | - | 100.00 | 8.60 | 846 | 13.6 | 8.0 | 236 |
| 12 | PV | 11.00 | *JAK2*V617F | 0.00533 | RUX | - | 100.00 | 26.67 | 474 | 14.2 | 8.0 | 294 |
| 13 | PV | 14.00 | *JAK2*V617F | 0.0195 | no cytoreductive therapy | - | 100.00 | 16.70 | 755 | 13.4 | 9.3 | 222 |
| 14 | PV | 16.00 | *JAK2V617F* | 0.00567 | IFNa | - | 100.00 | 8.33 | 440 | 9.5 | 3.4 | 178 |
| 15 | ET | 16.00 | *CALR*del52 | 0.00913 | IFNa | - | 86.67 | 4.00 | 340 | 14.8 | 6.5 | 158 |
| 16 | PMF | 16.00 | *JAK2*V617F | 0.009719 | no cytoreductive therapy | - | 96.67 | 10.30 | 581 | 13.9 | 5.9 | 235 |
| 17 | ET | 17.00 | *JAK2*V617F | 0.004902 | HU | - | 86.67 | 1.90 | n.a. | n.a. | n.a. | n.a. |
| 18 | ET | 17.00 | *CALRins5* | 0.00747 | IFNa | - | n.a. | n.a. | n.a. | n.a. | n.a. | n.a. |
|  | ET | 31.00 | *CALR*ins5 | 0.00853 | IFNa | - | 100.00 | 38.33 | 378 | 14.4 | 6.5 | 207 |
| 19 | ET | 17.00 | *JAK2V617F* | 0.017474 | others | - | 80.00 | 27.10 | 448 | 14.9 | 6.9 | 238 |
| 20 | ET | 18.00 | *CALR*del52 | 0.01063 | others | - | n.a. | n.a. | n.a. | n.a. | n.a. | n.a. |
| 21 | ET | 19.00 | *JAK2*V617F | 0.017 | IFNa | - | 100.00 | 15.00 | 348 | 12.4 | 3.7 | 151 |
| 22 | ET | 20.00 | *JAK2*V617F | 0.00357 | IFNa | - | 100.00 | 13.33 | 331 | 14.5 | 5.8 | 304 |
| 23 | PMF | 20.00 | *CALR*ins5 | 0.02025 | RUX | 17% NFE2 P236A, 14% TET2 G876*, 25% TET2 C1378Y | 100.00 | 35.00 | 1032 | 14.9 | 13.1 | 298 |
| 24 | PMF | 20.00 | *JAK2*V617F | 0.005746 | no cytoreductive therapy | - | 100.00 | 18.30 | 91 | 8.2 | 10.4 | 724 |
| 25 | ET | 21.00 | *JAK2*V617F | 0.001639 | others | - | 73.33 | 18.20 | 713 | 16.1 | 9.8 | 223 |
| 26 | PV | 22.00 | *JAK2*V617F | 0.024793 | no cytoreductive therapy | - | 96.67 | 29.31 | 443 | 13.1 | 7.2 | 281 |
| 27 | ET | 22.00 | *JAK2*V617F | 0.01013 | no cytoreductive therapy | - | 90.00 | 20.37 | 1160 | 13.3 | 8.4 | 247 |
| 28 | PV | 22.00 | *JAK2*V617F | 0.011063 | RUX | - | n.a. | n.a. | n.a. | n.a. | n.a. | n.a. |
| 29 | PV | 27.00 | *JAK2*V617F | 0.005224 | HU | - | 93.40 | 35.70 | 1224 | 12.7 | 11.3 | 329 |
| 30 | PV | 29.00 | *JAK2*V617F | 0.002827 | IFNa | - | 100.00 | 30.00 | 424 | 11.3 | 8.1 | 206 |
| 31 | PMF | 31.00 | *CALR*ins5 | 0.00954 | HU | - | 83.33 | 48.00 | 492 | 15.1 | 6.1 | 268 |
| 32 | ET | 35.00 | *CALR*del52 | 0.02063 | HU | - | 96.67 | 41.40 | 1552 | 13.5 | 7.6 | 292 |
| 33 | ET | 35.00 | *CALR*del49 | 0.00127 | IFNa | - | n.a. | n.a. | n.a. | n.a. | n.a. | n.a. |
| 34 | PMF | 37.00 | *CALR*del31 | 0.0044 | others | - | n.a. | n.a. | n.a. | n.a. | n.a. | n.a. |
| 35 | ET | 38.00 | *MPL*W515L | 0,007822508 | RUX | - | 83.33 | 20.00 | 776 | 12.8 | 6.5 | 312 |
| 36 | PMF | 40.00 | *JAK2*V617F | 0.004 | RUX | - | 84.00 | 19.00 | 81 | 8.2 | 4.8 | 480 |
| 37 | PMF | 41.00 | *CALR*del52 | 0.01972 | no cytoreductive therapy | - | 100.00 | 50.00 | 740 | 12.7 | 7.4 | 546 |
| 38 | ET | 42.00 | *CALR*ins5 | 0.0021 | HU | - | 90.00 | 44.40 | 299 | 11.3 | 4.0 | 251 |
| 39 | PMF | 45.00 | *MPL*W515K | 0,034513937 | no cytoreductive therapy | - | 96.00 | 45.80 | 545 | 12.8 | 13.0 | 669 |
| 40 | PMF | 48.00 | *JAK2*V617F | 0.0058 | others | 48% ASXL1 1Bp Dup (p.Thr880Asnfs*2); 47% U2AF1 Q157R; NF A4: 62% KRAS T58I, 47% TET2 1Bp Del (p.Glu1470Lysfs*101) | 96.67 | 46.55 | 8 | 6.2 | 1.9 | 126 |
| 41 | PV | 49.00 | *JAK2*V617F | 0.00183 | HU | 32% TET2 2-Bp Del (p.His974Glnfs*8) | 93.33 | 42.90 | 355 | 15.9 | 8.9. | 218 |
| 42 | PMF | 49.00 | *JAK2*V617F | 0.02214 | RUX | 48% ASXL1 C856*, 97% CBL C416Y, 53% DNMT3A R882H, 44% U2AF1 Q157R] | 88.89 | 38.11 | 318 | 7.0 | 77.3 | 696 |
| 43 | PMF | 49.00 | *JAK2*V617F | 0.061345 | RUX | 48% ASXL1 C856*, 97% CBL C416Y,  53% DNMT3A R882H, 44% U2AF1 Q157R] | n.a. | n.a. | n.a. | n.a. | n.a. | n.a. |
| 44 | Post-PV-MF | 51.00 | *JAK2*V617F | 0.05585 | RUX | - | 96.67 | 51.70 | 247 | 9.6 | 35.3 | 1070 |
| 45 | PV | 54.00 | *JAK2*V617F | 0.00732 | no cytoreductive therapy | - | 88.00 | 77.50 | 810 | 13.8 | 9.5 | 426 |
| 46 | ET | 55.00 | *CALR*del52 | 0.010166 | others | - | 90.00 | 37.00 | 361 | 13.1 | 7.3 | 247 |
| 47 | ET | 56.00 | *CALR*del52 | 0.0113 | RUX | - | 90.00 | 50.00 | 384 | 9.6 | 5.9 | 561 |
| 48 | PV | 56.00 | *JAK2*V617F | 0.0042 | IFNa | - | 100.00 | 60.00 | 1187 | 15.0 | 12.1 | 471 |
| 49 | PMF | 62.00 | *JAK2*V617F | 0.01993 | HU | - | 96.67 | 29.31 | 532 | 15.3 | 18.1 | 879 |
| 50 | PMF | 70.00 | *JAK2*V617F | 0.02413 | others | 51%SRSF2 24bp Del (P95 R102del) | n.a. | n.a. | n.a. | n.a. | n.a. | n.a. |
| 51 | PMF | 75.00 | *JAK2*V617F | 0.0191 | HU | 29% ASXL1c.1934dup (p.Gly646Trpfs*12) | n.a. | n.a. | n.a. | n.a. | n.a. | n.a. |
| 52 | PMF | 78.00 | *MPL*W515L | 0,04673036 | no cytoreductive therapy | - | 92.00 | 50.00 | 300 | 11.5 | 4.7 | 723 |
| 53 | PV | 80.00 | *JAK2*V617F | 0.01067 | RUX | - | 96.67 | 10.34 | 694 | 10.0 | 32.2 | 249 |
| 54 | PV | 81.00 | *JAK2*V617F | 0.014566 | RUX | - | 100.00 | 52.00 | 389 | 13.6 | 10.2 | 245 |
| 55 | Post PV MF | 88.00 | *JAK2*V617F | 0.03094 | no cytoreductive therapy | - | 74.40 | 71.43 | 536 | 14.0 | 11.8 | 640 |
| 56 | ET | 88.00 | *JAK2*V617F | 0.025907 | no cytoreductive therapy | - | 92.00 | 58.70 | 739 | 13.2 | 21.6 | 429 |
| 57 | PMF | 89.00 | *JAK2*V617F | 0.227972 | RUX | - | 93.33 | 85.71 | n.a. | n.a. | n.a. | n.a. |
| 58 | PV | 92.00 | *JAK2*V617F | 0.1836 | no cytoreductive therapy | - | 100.00 | 80.00 | 6 | 13.2 | 18.0 | 382 |
| 59 | PV | 94.00 | *JAK2*V617F | 0.0022 | HU | 32% TET2 T1397I | n.a. | n.a. | n.a. | n.a. | n.a. | n.a. |


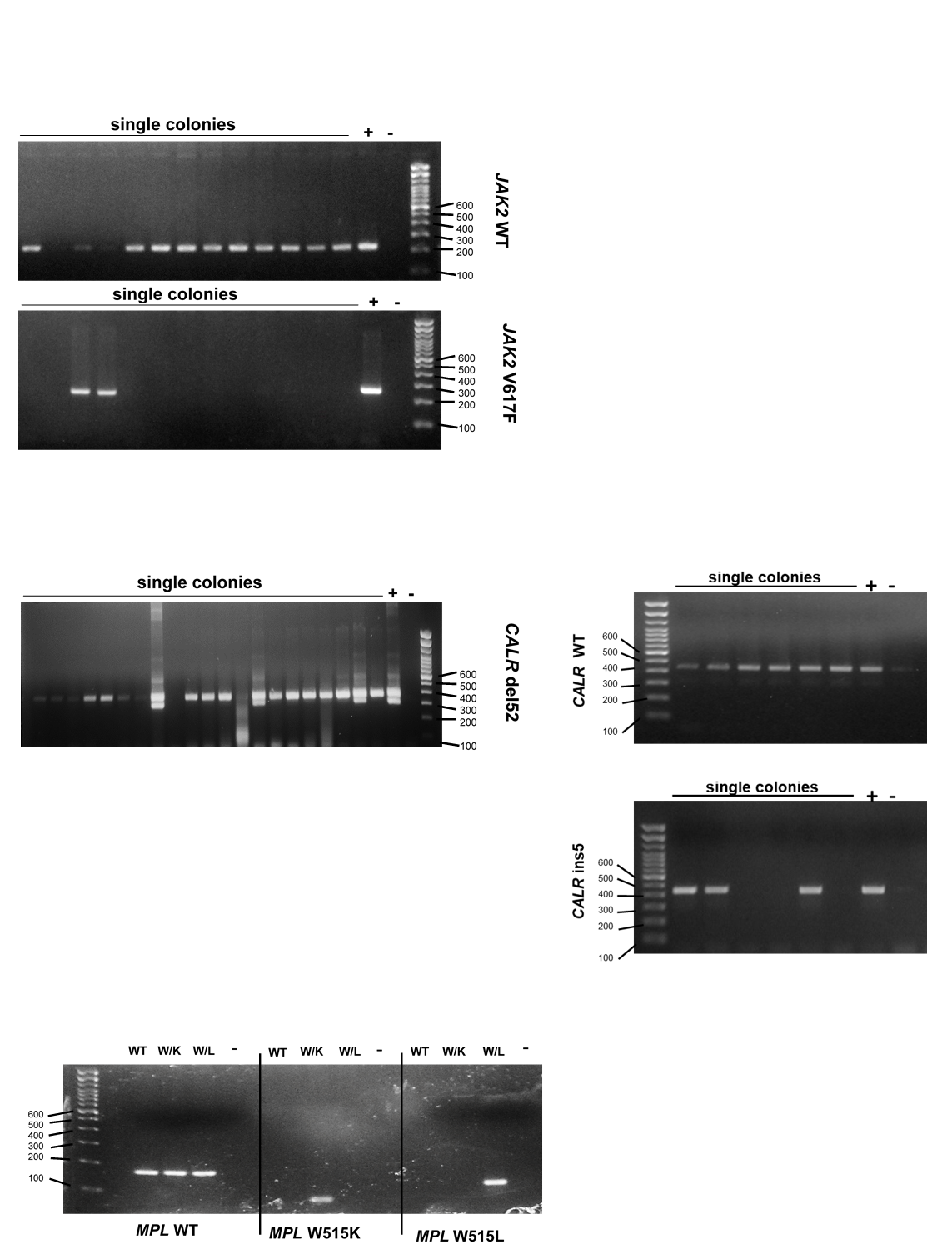


**a**

**d**

**b**

**c**

**Supplemental Fig. S1** Representative images of agarose gels used for genotyping. **a** Allele-specific PCRs for analysis of JAK2 genotype. Shown is analysis of colonies of one patient. **b** Analysis of deletions in CALR gene. PCR comprises the part of the gene, where deletions occur. Discrimination of mutated and WT due to fragment size. Shown are colonies from one patient with CALRdel52 mutation. **c** Allele-specific PCRs for CALRins5 mutation. Shown are colonies from one patient carrying CALRins5 mutation. **d** Analysis of common mutations in MPL gene. Shown are examples using DNA from patients carrying the respective mutations (found in NGS).

**
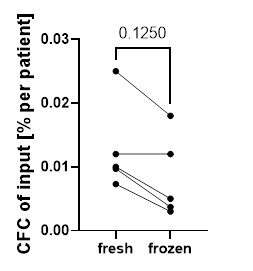
**

**Supplemental Fig S2** Cloning efficiency was slightly but not significantly decreased in frozen samples. PBMCs from patients from the same date were seeded in CFU assays before and after being frozen. % CFC was calculated and compared in fresh and frozen samples. Significance was analyzed using Wilcoxon-Test.

**b**

**a**

**
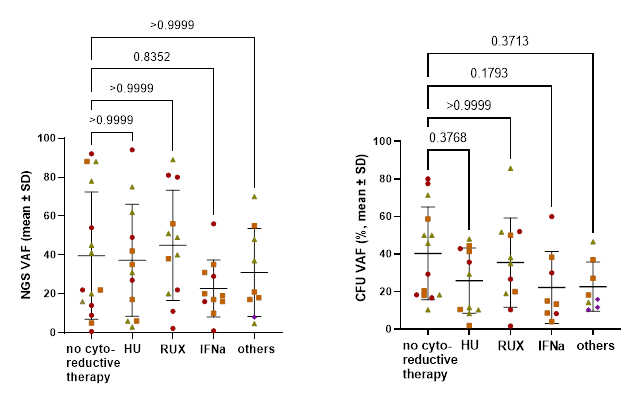
**

**Supplemental Fig. S3** Mutant VAF was compared in different treatment groups. **a** NGS VAF in the different groups is shown. **b** CFU-determined VAF is displayed. Statistical analysis was done using Kruskal-Wallis test with Dunn’s multiple comparison. No cytoreductive therapy – no treatment, phlebotomies and/or ASS; HU – hydroxyurea; RUX – ruxolitinib; IFNa – (pegylated) Interferon alpha; others – e.g. Imetelstat, Anagrelide or combination treatment. Statistical analysis was done using Kruskal-Wallis test with Dunn’s multiple comparison. red dots – PV; orange squares – ET; green triangles – MF, violet hashes - MPN-unclassifiable


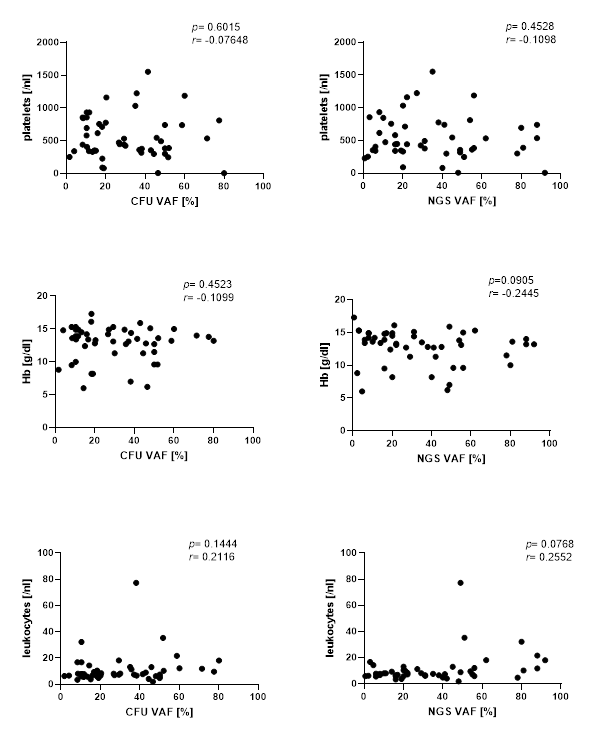


**e**

**f**

**d**

**c**

**b**

**a**

**Supplemental Fig. S4 Correlations of different blood parameters with the mutant VAF. a and b** Number of platelets was correlated with VAF. No correlations with CFU (**a**) or NGS VAF (**b**) were found. **c and d** Hemoglobin levels were correlated with VAF. No correlations with CFU (**c**) or NGS (**d**) VAF were found. **e and f** Number of leukocytes was correlated with mutant VAF. No correlation with CFU VAF (**e**) or NGS VAF (**f**) was found.
